# Notice of Memorials Concerning the Treatment of Pulmonary Phthisis, and the Physiological and Therapeutical Action of the Hypophosphites

**Published:** 1859-09

**Authors:** J. Francis Churchill


*Notice of Memorials concerning the Treatment of Pulmonary Phthisis, and the Physiological and Therapeutical Action of the Hypophosphites.* By J. Francis Churchill. *Presented to the Imperial Academy of Sciences.*

There is no disease in the long list that human nature is subject to, the treatment of which has occupied the minds of physicians so constantly and for so long a period as that of pulmonary phthisis. Time after time the discoveries of remedies have been announced. The compounds of iron, sulphur, iodine, cod-liver oil, etc., have been arrayed before the public and had fair and persistent trials, and they all have failed to establish the virtues claimed for them, although we admit that many of them are exceedingly useful in palliating some of the distressing symptoms of this disease.

At this point we find Dr. J. Francis Churchill, a physician well known to the medical profession, taking up the study of tuberculosis in 1855, while engaged in the practice of his profession in Havana. Dr. Churchill, in his report, says:

"The first idea I had upon this subject—that which induced me to investigate it—and which, I conceive, furnishes a clue to that mysteriously morbid condition entitled tuberculosis, dates from the month of February, 1855, while I was engaged in the practice of my profession in Havana.

"Occupied as I have been since the commencement of my medical career, in researches upon the treatment of phthisis, my investigations led me to believe that the tubercular diathesis depended wholly upon the disturbance of the primordial functions of life; that considering the intimate union that exists between all parts of the body, this disturbance must have, as a proximate cause, some promitant modification in the process of sanguification. The works of various pathologists, especially those of MM. Andral and Gavarret, the correctness of which has since been confirmed by others, indicated that the variation in the composition of the blood, in pulmonary phthisis, had no peculiar and important relation, as far as its organic elements were concerned. I was, consequently, induced to believe that it was among the inorganic elements of this fluid that we should look for the *special cause* of this diathesis.

"Medical science furnishes a numerous collection of data going to show the influence upon the economy of the greater portion of these chemical elements: as, for instance, iron, sulphur, (whether in the state of a sulphuret or as a sulphate,) and the chlorides. The use of alkaline remedies was an every day occurrence—all, or nearly all, had been tried in the treatment of tuberculosis; and although each one of them, in its turn, had been extolled, neither of these agents had presented sufficiently well marked, or uniform effects, to establish its claim as possessing a real curative influence over the disease. I therefore deducted these substances from those with which I designed to experiment, intending subsequently to take them up should I find it necessary. I decided to commence my investigations by the use of phosphorus."

Dr. Churchill next reviews the use of the phosphates and phosphorus, both of which had been recommended as specific in tubercular diathesis, and accounts for their failure on the hypothesis—

"That the phosphorized element of the blood, or of organic matter, exists in the body in some other form than that of phosphoric acid; it was evident to me that it performed the double part of an eminently combustible substance, and at the same time entered into molecular combination with the other elements of the body, in such a way as to become an integral portion of it. But neither phosphoric acid nor phosphorus can fulfil this double indication. The former is already at its maximum of oxydation; the latter placed in contact with the liquids of the stomach, or with any of the tissues of the body, must necessarily, before being absorbed for assimilation, become transformed into phosphoric acid, or the phosphorus a hypophosphorus acid: compounds next lower on the degree of oxydation.

"These reasons explain, to my mind, the causes why the preparations of phosphorus have proved so untrustworthy; and why, on the one hand, Coindet was enabled to obtain certain satisfactory results upon employing it in large doses, while MM. Barthez and Rilliet, with much less doses, obtained only unfavorable or negative ones.

"Being satisfied that I have discovered the reason for these apparent contradictions, I believe I can establish the following double hypothesis:

"The tubercular diathesis depends upon a diminution in the system of the phosphorized element, and which, having to act the part of a combustible body, should be found at a lower degree of oxydation than that of phosphoric acid.

"After I had formed this mental induction nothing remained for me but to verify it by facts, and to choose which, among the various combinations of phosphorus with oxygen, viz., the oxide of phosphorus, the hypophosphorus, or the phosphorus acid, I should use. My decision was based upon the following considerations:

"The red oxide of phosphorus is insoluble and very inflammable; the yellow variety, although soluble, forms combinations with bases which are very slightly so, and are by no means permanent. In addition to this, there was an objection in the difficulty of its preparation under the circumstances in which I was then placed.

"I judged that phosphorus acid would have an action much less energetic than hypophosphorus acid, it being less combustible, since it contains three equivalents of oxygen, while the latter is combined with one equivalent only. The salts formed from the former, which are soluble, are those of soda, potassa, ammonia, while *all the hypophosphites are soluble in water*. I, therefore, chose the hypophosphorus acid, and decided to use it combined with a base. The use of the acid itself, uncombined, seemed to me to offer no particular advantage, since at the moment it is absorbed it enters into chemical combination with the alkaline carbonates of the blood. There was also the additional objection that it was difficult to divide it into doses; for, if used it would be necessary to determine, each time, the degree of concentration of the acid. From preference I chose to commence my experimentation with the hypophosphite of lime, on account of the important part which this base is supposed to play in the system."

Upon this theory the practice is proceeded with, and hypophosphites of various bases are employed. Dr. Churchill, in his first report, gives very detailed statements of thirty four cases. Nine of these cases were thoroughly cured, eleven relieved, and fourteen appeared to receive no benefit. If these relative numbers are to express what future practice is to be in the use of the hypophosphites, these compounds would certainly be said to possess great virtue.

In a subsequent report by Dr. Churchill, made to the Imperial Academy of Sciences at Paris, he says:

"I have the honor to submit for the consideration of the 'Academy' several memoranda, giving the results of the treatment of forty one cases of phthisis, by the hypophosphites, since the publication of my work, a copy of which I now lay before it. These results *fully confirm all that I have heretofore written concerning the efficacy of these preparations in pulmonary phthisis:* and it would be easy for me to show that the ill success of other practitioners, in such cases, was owing either, 1st, To the leisons pre-existing to the treatment which were of themselves sufficient to cause death; 2d, To the existence of complications; or, 3d, To the impurity of the salts employed, and which were administered without regard to the conditions I have laid down and consider essential.

"I have no hesitation in saying, that when these conditions are complied with, *the cure of consumption in the second and third stages* (at a period, therefore, when there can be no doubt as to the nature of the disease,) *is the rule, while death is the exception.* I am also prepared to assert that, contrary to the opinions generally received, the third stage of consumption is, all other circumstances being equal, more amenable to treatment than the second. Hereditary predisposition seems in no way to counteract the curative powers of the hypophosphites, as the patients in whom it was most strongly marked, recovered as rapidly as the others. I, therefore, ask the opinion of this Academy upon the cases of disease of which I now present the notes of observation (made before any decided result was observed), in order that it may be able to determine whether the cases in question were actually attacked with pulmonary phthisis.

"It is not alone as a *curative agent*, but above all as a *prophylactic*, that the hypophosphites should be employed in combating a disease which (as M. Payer has shown) is almost entirely unknown among nations in a savage state, but which has become the permanent scourge of civilized life."

In regard to the manner of administering it, we cannot do better than extract from a letter of Dr. Churchill on the subject in the London Medical Circular:

"Sir: In reply to the inquiry of your correspondent, Dr. W. J.,' I beg to inform your readers that the dose of the hypophosphite which I have found the most manageable is ten grains at first, increasing it gradually up to one scruple daily. This quantity I seldom exceed, though in some cases I have used larger doses with benefit. Children, under four years of age, can seldom take more than from one fifth to two fifths of a grain daily. In all cases, however, with this as with any other remedy, the physician must watch its effects upon the system, which vary with the idiosyncrasy of the individual. The hypophosphite of soda having, *when pure*, nearly the same taste as common salt, may be given in any form. I usually prescribe each dose to be taken in a tumbler full of sweetened water or sweetened milk, or wine and water, or both, or any other drink that can be taken at breakfast or dinner. I use no other treatment of any kind unless required by the existence of complications, such as intercurrent inflammation of the lungs, diarrhœa, cardiac disease, etc."

With these facts before us, it certainly becomes the medical profession to give the hypophosphites a fair and persistent trial, whether or not we lay any stress on Dr. Churchill's theory. The compounds and pharmaceutical preparations of hypophosphorus that have been recommended and employed are—

| | |
|---|---|
| Hypophosphite of Lime,* | Hypophosphite of Soda, |
| " " Potash, | " " Ammonia, |
| " " Iron, | " " Manganese, |
| " " Quinine. | |

Also syrups containing one or more of these salts. The last salt mentioned, namely, the hypophosphite of quinine, was recommended a short time since by Prof. J. Lawrence Smith. We have since ascertained that Dr. Churchill had recommended it before, although the salt prepared for him under that name must have been a very different looking compound from the one prepared by Dr. Smith.—*Louisville Med. News.*

---

*We take pleasure in informing our readers that we have been in the habit of using these compounds, as manufactured at the Louisville Chemical Works, and can recommend them to the profession, both for their purity and beauty. The advertisement of these works can be seen on the third page of our cover.—Editors.

